# Metagenomic Sequencing of Monkeypox Virus, Northern Mexico

**DOI:** 10.3201/eid2902.221199

**Published:** 2023-02

**Authors:** Kame A. Galán-Huerta, Manuel Paz-Infanzon, Laura Nuzzolo-Shihadeh, Alí F. Ruiz-Higareda, Paola Bocanegra-Ibarias, Daniel Z. Villareal-Martínez, Fania Z. Muñoz-Garza, Maria D. Guerrero-Putz, Barbara Sáenz-Ibarra, Oralia Barboza-Quintana, Jorge Ocampo-Candiani, Ana M. Rivas-Estilla, Adrian Camacho-Ortiz

**Affiliations:** Universidad Autonónoma de Nuevo León, Monterrey, Mexico

**Keywords:** Monkeypox virus, mpox, Mexico, phylogeny, sexually transmitted infections, viruses

## Abstract

Monkeypox virus (MPXV) has gained interest because of a multicountry outbreak of mpox (formerly monkeypox) cases with no epidemiologic link to MPXV-endemic regions. We sequenced the complete genome of MPXV isolated from a patient in northern Mexico. Phylogenetic analysis grouped the virus with isolates from Germany.

Monkeypox virus (MPXV) is a zoonotic pathogen that causes mpox (formerly monkeypox), a febrile rash disease, in humans. It has caused multiple outbreaks in the past (*1*) but recently acquired international attention because of a multicountry outbreak of mpox cases with no epidemiologic link to MPXV-endemic regions (*2*). During January–June 2022, a total of 3,413 laboratory-confirmed cases and 1 death were reported to the World Health Organization (*3*).

MPXV is a double-stranded DNA virus that belongs to the genus *Orthopoxvirus* within the Poxviridae family. A total of 3 clades have been proposed: clades 1, 2, and 3 (*4*). Genomes belonging to the recent outbreaks gather at clade 3 and create the human MPXV1 subclade.

As of July 4, 2022, Mexico had 27 confirmed mpox infections (*5*). On June 28, 2022, a 34-year-old man with HIV sought care at the Dermatology Service of the Hospital Universitario Dr. José Eleuterio González (Monterrey, Mexico). The patient had multiple 1–2-cm flesh-colored papules with ulcerated centers and elevated borders on the dorsal area of the penis and the groin area and had bilateral inguinal lymphadenopathy. He had engaged in multiple high-risk sexual encounters during the previous several months and had traveled to Mexico City and Guadalajara. We collected skin swab samples from the lesions and extracted DNA by using the High Pure PCR Template Preparation Kit (Roche, https://www.roche.com) in a biological safety cabinet. We disposed of hazardous biological infectious waste according to NOM-087-ECOL-SSA1–2002 and Centers for Disease Control and Prevention regulations. We used quantitative PCR to detect viral DNA as described in Li et al. (*6*). The study was approved by the ethics committee of the Hospital Universitario Dr. José Eleuterio González (registration no. IF22-00006). Written informed consent was obtained from the patient.

We performed genome sequencing at the Medical Virology Research and Innovation Center (Monterrey, Mexico). We used 700 ng of DNA to prepare the library with the Ligation Sequencing Kit (Oxford Nanopore Technologies, https://nanoporetech.com), by duplicate, following the manufacturer’s protocol. We loaded libraries to a FLO-MIN106 R9.4.1. on a MinION device (Oxford Nanopore Technologies). We used high-accuracy basecalling and depleted the human genome by adaptive sampling reference (GRCh38.p14, GCF_000001405.40) in MinKNOW version 22.05.5 (Oxford Nanopore Technologies).

We assembled the genome with Minimap2 version 2.17, using an MPXV reference genome (GenBank accession no. NC_063383.1), and consensus sequence was obtained with Medaka version 1.0.3 (https://pypi.org/project/medaka/1.0.3) and bcftools version 1.10.12 (https://github.com/samtools/bcftools/releases). We deposited the obtained sequence in GISAID (accession no. EPI_ISL_13607904) and GenBank (accession no. ON911481). Furthermore, raw reads were mapped against the human genome and filtered out using Minimap2 and Samtools version 1.16 (http://www.htslib.org). Remaining reads were assembled de novo with Flye version 2.9.1 (https://anaconda.org/bioconda/flye) and subsequently polished using Medaka and homopolish version 0.4 (https://anaconda.org/bioconda/homopolish/files).

We used Nextstrain’s mpox bioinformatic processing workflow (*7*) for our initial analysis. Subsequently, we downloaded 399 sequences from GenBank that belonged to clade hMPXV1 from outbreaks in 2017–2019 and 2022 (the available sequences as of July 14, 2022).

We masked and aligned sequences in MAFFT version 7.505 (https://mafft.cbrc.jp/alignment/software). We inferred a maximum-likelihood phylogenetic tree with IQ-TREE version 2.0.3 (http://www.iqtree.org) using the Hasegawa-Kishino-Yano plus base frequencies plus invariant sites substitution model with 1,000 replicates for SH-like approximate likelihood ratio test and 1,000 replicates for ultrafast bootstrap. 

Adding both sequencing runs, we obtained 1.77 million reads with an estimated N50 of 17.2 kb after a 20-h run. The MPXV genome sequence obtained by reference assembly was composed of 55,512 mapped reads with a breadth of 197,414 bp, 751× average depth, and 1,212× maximum depth. The sequence obtained by de novo assembly was composed of 58,259 mapped reads with a breadth 197,875 bp, 752× average depth, and 1,215× maximum depth.

Phylogenetic analyses revealed that the obtained sequence clusters within the newly proposed B.1 lineage from clade 3, where sequences from the multicountry mpox outbreak are located (Figure, panel A; Appendix Figure 1, panel A). Sequences from Europe (specifically Portugal and Germany) dominate the tree because they make up most of the available sequences. The Mexico sequence groups in a well-supported clade with sequences from Germany (Figure, panel B; Appendix Figure 1, panel B), although we could not establish an epidemiologic relationship. The sequence is grouped in the same clade in both approaches, independent of the assembly method (Appendix Figure 2). The inferred date of the most recent common ancestor of the clade is May 26, 2022 (95% CI May 26, 2022–June 2, 2022). Sequences from this clade share the mutation G120241A, causing a nonsynonymous substitution in OPG137, D214N. OPG137 encodes for a virion membrane assembly protein, which is involved in the crescent membrane and immature virion formation (*8*). Aspartic acid is an acidic amino acid and asparagine is neutral. The effect of this mutation has yet to be determined.

**Figure:**
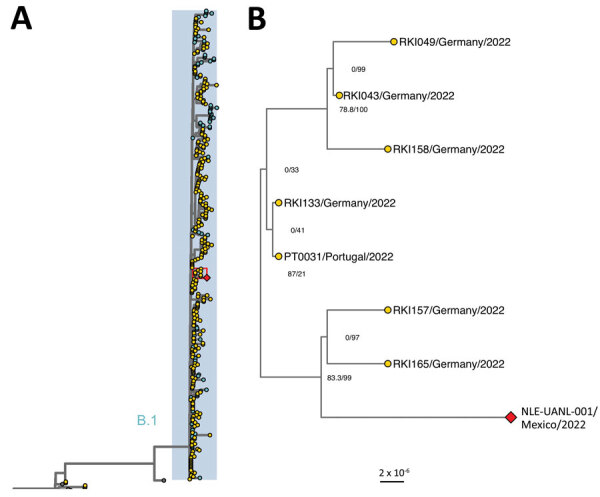
Phylogenetic relationship of monkeypox virus genomes, 2017–2022. A) Maximum-likelihood phylogenetic tree with 400 whole-genome sequences of monkeypox virus made in IQTree (http://www.iqtree.org). Highlighted genomes in blue belong to the proposed B.1 lineage. B) Magnification of area in red box in panel A showing clade indicating the Mexico isolate and related sequences. Node numbers indicate SH-like approximate likelihood ratio test and ultrafast bootstrap values. The red diamond corresponds to the sequence from this study and yellow circles to sequences from Europe. The list of accession numbers of the sequences is shown in the Appendix Table. The tree was annotated in ggtree (https://bioconductor.org/packages/release/bioc/html/ggtree.html). Scale bar indicates substitutions per site.

In conclusion, we sequenced the whole genome of MXPV from a patient with mpox in northern Mexico. Subsequent sequencing will unravel how the virus is transmitting and adapting throughout the population. According to our results, this patient likely had close contact with an infected person who traveled from Europe.

Appendix Additional information about metagenomic sequencing of monkeypox virus case, northern Mexico
