# Supplementary material for: Metagenomic Sequencing of Monkeypox Virus, Northern Mexico
Source: Emerg Infect Dis. 2023 Feb;29(2):448–50. doi: 10.3201/eid2902.221199 (PMC9881790; doi:10.3201/eid2902.221199)
Supplement: Appendix — Additional information about metagenomic sequencing of monkeypox virus case, northern Mexico [file 22-1199-Techapp-s1.pdf]

Article DOI: https://doi.org/10.3201/eid2902.221199

Metagenomic Sequencing of Monkeypox Virus Case, Northern Mexico

Appendix

Appendix Table. NCBI Genbank dataset used in this study

Accession number	Country	Lineage	Collection date	Reference
KJ642615.1	Nigeria	NA	1978	Nakazawa,Y., 2014
KJ642617.1	Nigeria	NA	1971	Nakazawa,Y., 2014
MG693723.1	Nigeria	A	2017–12–31	Faye,O. 2017
MG693724.1	Nigeria	A	2017	Faye,O. 2017
MK783027.1	Nigeria	A	2017	Yinka-Ogunleye,A. 2019
MK783028.1	Nigeria	A	2017–11–09	Yinka-Ogunleye,A. 2019
MK783029.1	Nigeria	A	2017–12–06	Yinka-Ogunleye,A. 2019
MK783030.1	Nigeria	A	2017–11–30	Yinka-Ogunleye,A. 2019
MK783031.1	Nigeria	A	2017–11–09	Yinka-Ogunleye,A. 2019
MK783032.1	Nigeria	A	2017–11	Yinka-Ogunleye,A. 2019
MK783033.1	Nigeria	A	2017	Yinka-Ogunleye,A. 2019
MN648051.1	Israel	A.1	2018–10–04	Cohen Gihon,I. 2019
MT250197.1	Singapore	A.1	2019	Yong,SEF. 2020
MT903337.1	Nigeria	A	2018	Mauldin,M.R. 2020
MT903338.1	Nigeria	A	2018	Mauldin,M.R. 2020
MT903339.1	Nigeria	A	2018	Mauldin,M.R. 2020
MT903340.1	Nigeria	A	2018	Mauldin,M.R. 2020
MT903341.1	Nigeria	A.1	2018–08–14	Mauldin,M.R. 2020
MT903342.1	Singapore	A.1	2019–04–30	Mauldin,M.R. 2020
MT903343.1	UK	A.1	2018	Mauldin,M.R. 2020
MT903344.1	UK	A.1	2018	Mauldin,M.R. 2020
MT903345.1	UK	A.1	2018	Mauldin,M.R. 2020
NC_063383.1	Nigeria	A	2018–08–01	Mauldin,M.R. 2022
ON563414.3	USA	B.1	2022–05	Gigante,C.M. 2022
ON568298.1	Germany	B.1	2022–05–19	Antwerpen,M.H. 2022
ON585029.1	Portugal	B.1	2022–05–04	Isidro,J. 2022
ON585030.1	Portugal	B.1	2022–05–15	Isidro,J. 2022
ON585031.1	Portugal	B.1	2022–05–15	Isidro,J. 2022
ON585032.1	Portugal	B.1	2022–05–17	Isidro,J. 2022
ON585033.1	Portugal	B.1	2022–05–15	Isidro,J. 2022
ON585034.1	Portugal	B.1	2022–05–15	Isidro,J. 2022
ON585035.1	Portugal	B.1	2022–05–15	Isidro,J. 2022
ON585036.1	Portugal	B.1	2022–05–15	Isidro,J. 2022
ON585037.1	Portugal	B.1	2022–05–15	Isidro,J. 2022
ON585038.1	Portugal	B.1	2022–05–15	Isidro,J. 2022
ON595760.2	Switzerland	B.1	2022–05–19	Laubscher,F. 2022
ON602722.2	France	B.1	2022–05	Croville,G. 2022
ON609725.2	Slovenia	B.1	2022–05–23	Zakotnik,S. 2022
ON614676.1	Italy	B.1	2022–05–18	Gruber,C.E.M. 2022
ON615424.1	Netherlands	B.1	2022–05	Oude Munnink,B.B. 2022
ON619835.2	UK	B.1	2022–05	Groves,N. 2022
ON619836.2	UK	B.1	2022–05	Groves,N. 2022
ON619837.2	UK	B.1	2022–05	Groves,N. 2022
ON619838.2	UK	B.1	2022–05	Groves,N. 2022
ON622712.1	Belgium	B.1	2022–05–19	Vanmechelen,B. 2022
ON622713.1	Belgium	B.1	2022–05–22	Wawina-Bokalanga,T. 2022
ON622718.1	Spain	B.1	2022–05	Martinez-Puchol,S. 2022
ON622720.1	Switzerland	B.1	2022–05–24	Laubscher,F. 2022
ON622722.2	France	B.1	2022–05–22	2022
ON627808.1	USA	B.1	2022–05–20	Young,E.L. 2022
ON631241.1	Slovenia	B.1	2022–05–24	Zakotnik,S. 2022
ON631963.1	Australia	B.1	2022–05	Hammerschlag,Y. 2022
ON637938.1	Germany	B.1	2022–05	Brinkmann,A. 2022
ON637939.1	Germany	B.1	2022–05	Brinkmann,A. 2022

Accession number	Country	Lineage	Collection date	Reference
ON644344.1	Italy	B.1	2022–05–25	Licastro,D. 2022
ON645312.1	UK	B.1	2022–05–11	Alcolea-Medina,A. 2022
ON649708.1	Portugal	B.1	2022–05–19	Isidro,J. 2022
ON649709.1	Portugal	B.1	2022–05–18	Isidro,J. 2022
ON649710.1	Portugal	B.1	2022–05–18	Isidro,J. 2022
ON649711.1	Portugal	B.1	2022–05–18	Isidro,J. 2022
ON649712.1	Portugal	B.1	2022–05–19	Isidro,J. 2022
ON649713.1	Portugal	B.1	2022–05–19	Isidro,J. 2022
ON649714.1	Portugal	B.1	2022–05–20	Isidro,J. 2022
ON649715.1	Portugal	B.1	2022–05–20	Isidro,J. 2022
ON649716.1	Portugal	B.1	2022–05–23	Isidro,J. 2022
ON649717.1	Portugal	B.1	2022–05–21	Isidro,J. 2022
ON649718.1	Portugal	B.1	2022–05–20	Isidro,J. 2022
ON649719.1	Portugal	B.1	2022–05–23	Isidro,J. 2022
ON649720.1	Portugal	B.1	2022–05–19	Isidro,J. 2022
ON649721.1	Portugal	B.1	2022–05–19	Isidro,J. 2022
ON649722.1	Portugal	B.1	2022–05–19	Isidro,J. 2022
ON649723.1	Portugal	B.1	2022–05–20	Isidro,J. 2022
ON649724.1	Portugal	B.1	2022–05–23	Isidro,J. 2022
ON649725.1	Portugal	B.1	2022–05–23	Isidro,J. 2022
ON649879.1	Israel	B.1	2022–05–20	Israeli,O. 2022
ON674051.1	USA	A.2	2022–05	Gigante,C.M. 2022
ON675438.1	USA	A.2	2022–05	Gigante,C.M. 2022
ON676703.1	USA	B.1	2022–05	Gigante,C.M. 2022
ON676704.1	USA	B.1	2022–05	Gigante,C.M. 2022
ON676705.1	USA	B.1	2022–05	Gigante,C.M. 2022
ON676706.1	USA	B.1	2022–05	Gigante,C.M. 2022
ON676707.1	USA	A.2	2021–07	Gigante,C.M. 2022
ON676708.1	USA	A.1.1	2021–11	Gigante,C.M. 2022
ON682263.3	Germany	B.1	2022–05–04	Brinkmann,A. 2022
ON682264.3	Germany	B.1	2022–05–31	Brinkmann,A. 2022
ON682265.3	Germany	B.1	2022–05–15	Brinkmann,A. 2022
ON682266.2	Germany	B.1	2022–05–04	Brinkmann,A. 2022
ON682267.2	Germany	B.1	2022–05–01	Brinkmann,A. 2022
ON682268.2	Germany	B.1	2022–05–04	Brinkmann,A. 2022
ON682269.3	Germany	B.1	2022–05–01	Brinkmann,A. 2022
ON682270.2	Germany	B.1	2022–05–01	Brinkmann,A. 2022
ON694329.1	Germany	B.1	2022–05–15	Brinkmann,A. 2022
ON694330.1	Germany	B.1	2022–05–31	Brinkmann,A. 2022
ON694331.1	Germany	B.1	2022–05–18	Brinkmann,A. 2022
ON694332.1	Germany	B.1	2022–05–31	Brinkmann,A. 2022
ON694333.1	Germany	B.1	2022–05–15	Brinkmann,A. 2022
ON694334.1	Germany	B.1	2022–05–31	Brinkmann,A. 2022
ON694335.1	Germany	B.1	2022–05–23	Brinkmann,A. 2022
ON694336.1	Germany	B.1	2022–05–01	Brinkmann,A. 2022
ON694337.1	Germany	B.1	2022–05–31	Brinkmann,A. 2022
ON694338.1	Germany	B.1	2022–05–15	Brinkmann,A. 2022
ON694339.1	Germany	B.1	2022–05–18	Brinkmann,A. 2022
ON694340.1	Germany	B.1	2022–05–31	Brinkmann,A. 2022
ON694341.1	Germany	B.1	2022–05–04	Brinkmann,A. 2022
ON694342.1	Germany	B.1	2022–05–17	Brinkmann,A. 2022
ON720848.1	Spain	B.1	2022–05–27	Buenestado-Serrano,S. 2022
ON720849.1	Spain	B.1	2022–05–27	Buenestado-Serrano,S. 2022
ON736420.1	Canada	B.1	2022–05–31	Croxen,M. 2022
ON745215.1	Italy	B.1	2022–05–19	Giombini,E. 2022
ON745225.1	Spain	B.1	2022–05–27	Buenestado-Serrano,S. 2022
ON751962.1	Brazil	B.1	2022–06–07	Claro,I.M. 2022
ON754984.1	Slovenia	B.1	2022–06–01	Zakotnik,S. 2022
ON754985.1	Slovenia	B.1	2022–06–01	Zakotnik,S. 2022
ON754986.1	Slovenia	B.1	2022–06–01	Zakotnik,S. 2022
ON754987.1	Slovenia	B.1	2022–05–23	Zakotnik,S. 2022
ON754989.1	Canada	B.1	2022–06–06	Croxen,M. 2022
ON755039.1	France	B.1	2022–05–19	Jarjaval,F. 2022
ON755040.1	France	B.1	2022–05–20	Jarjaval,F. 2022
ON755231.1	Germany	B.1	2022–06–01	Brinkmann,A. 2022
ON755232.1	Germany	B.1	2022–06–01	Brinkmann,A. 2022
ON755233.1	Germany	B.1	2022–06–01	Brinkmann,A. 2022
ON755234.1	Germany	B.1	2022–06–01	Brinkmann,A. 2022
ON755235.1	Germany	B.1	2022–06–01	Brinkmann,A. 2022

Accession number	Country	Lineage	Collection date	Reference
ON755236.1	Germany	B.1	2022–06–01	Brinkmann,A. 2022
ON755237.1	Germany	B.1	2022–06–01	Brinkmann,A. 2022
ON755238.1	Germany	B.1	2022–06–15	Brinkmann,A. 2022
ON755239.1	Germany	B.1	2022–06–15	Brinkmann,A. 2022
ON755240.1	Germany	B.1	2022–06–15	Brinkmann,A. 2022
ON755241.1	Germany	B.1	2022–06–01	Brinkmann,A. 2022
ON755242.1	Germany	B.1	2022–06–01	Brinkmann,A. 2022
ON755243.1	Germany	B.1	2022–06–15	Brinkmann,A. 2022
ON755244.1	Germany	B.1	2022–06–15	Brinkmann,A. 2022
ON755245.1	Germany	B.1	2022–06–01	Brinkmann,A. 2022
ON755246.1	Germany	B.1	2022–06–01	Brinkmann,A. 2022
ON755247.1	Germany	B.1	2022–06–15	Brinkmann,A. 2022
ON755248.1	Germany	B.1	2022–06–01	Brinkmann,A. 2022
ON755249.1	Germany	B.1	2022–06–15	Brinkmann,A. 2022
ON755250.1	Germany	B.1	2022–06–01	Brinkmann,A. 2022
ON755251.1	Germany	B.1	2022–06–15	Brinkmann,A. 2022
ON755252.1	Germany	B.1	2022–06–15	Brinkmann,A. 2022
ON755253.1	Germany	B.1	2022–06–01	Brinkmann,A. 2022
ON755254.1	Germany	B.1	2022–06–15	Brinkmann,A. 2022
ON755255.1	Germany	B.1	2022–06–15	Brinkmann,A. 2022
ON755256.1	Germany	B.1	2022–06–15	Brinkmann,A. 2022
OL504741.1	UK	A.1	2019–12	Atkinson,B. 2022
OL504742.1	UK	A.1	2019–12	Atkinson,B. 2022
OL504743.1	UK	A.1	2019–12	Atkinson,B. 2022
ON780016.1	Italy	B.1	2022–05–19	Rueca,M. 2022
ON780017.1	Italy	B.1	2022–05–21	Gramigna,G. 2022
ON782021.1	Finland	B.1	2022–05–24	Kant,R. 2022
ON782022.1	Finland	B.1	2022–05–31	Kant,R. 2022
ON782054.1	Spain	B.1	2022–05–31	Alcoba-Florez,J. 2022
ON782055.1	Spain	B.1	2022–05–31	Alcoba-Florez,J. 2022
ON792320.1	Switzerland	B.1	2022–06–01	Kufner,V. 2022
ON792321.1	Switzerland	B.1	2022–06–02	Kufner,V. 2022
ON792322.1	Switzerland	B.1	2022–06–02	Kufner,V. 2022
ON803413.1	Canada	B.1	2022–05–21	Knox,N. 2022
ON803414.1	Canada	B.1	2022–05–21	Knox,N. 2022
ON803415.1	Canada	B.1	2022–05–20	Knox,N. 2022
ON803416.1	Canada	B.1	2022–05–17	Knox,N. 2022
ON803417.1	Canada	B.1	2022–05–17	Knox,N. 2022
ON803418.1	Canada	B.1	2022–05–18	Knox,N. 2022
ON803419.1	Canada	B.1	2022–05–14	Knox,N. 2022
ON803420.1	Canada	B.1	2022–05–19	Knox,N. 2022
ON803421.1	Canada	B.1	2022–05–19	Knox,N. 2022
ON803422.1	Canada	B.1	2022–05–19	Knox,N. 2022
ON803423.1	Canada	B.1	2022–05–19	Knox,N. 2022
ON803424.1	Canada	B.1	2022–05–19	Knox,N. 2022
ON803425.1	Canada	B.1	2022–05–19	Knox,N. 2022
ON803426.1	Canada	B.1	2022–05–19	Knox,N. 2022
ON803427.1	Canada	B.1	2022–05–18	Knox,N. 2022
ON803428.1	Canada	B.1	2022–05–21	Knox,N. 2022
ON803429.1	Canada	B.1	2022–05–19	Knox,N. 2022
ON803430.1	Canada	B.1	2022–05–19	Knox,N. 2022
ON803431.1	Canada	B.1	2022–05–20	Knox,N. 2022
ON803432.1	Canada	B.1	2022–05–20	Knox,N. 2022
ON803433.1	Canada	B.1	2022–05–20	Knox,N. 2022
ON803434.1	Canada	B.1	2022–05–20	Knox,N. 2022
ON803435.1	Canada	B.1	2022–05–20	Knox,N. 2022
ON803436.1	Canada	B.1	2022–05–20	Knox,N. 2022
ON803437.1	Canada	B.1	2022–05–20	Knox,N. 2022
ON803438.1	Canada	B.1	2022–05–20	Knox,N. 2022
ON803439.1	Canada	B.1	2022–05–20	Knox,N. 2022
ON803440.1	Canada	B.1	2022–05–22	Knox,N. 2022
ON803441.1	Canada	B.1	2022–05–23	Knox,N. 2022
ON803442.1	Canada	B.1	2022–05–16	Knox,N. 2022
ON803443.1	Canada	B.1	2022–05–16	Knox,N. 2022
ON803444.1	Canada	B.1	2022–05–24	Knox,N. 2022
ON808413.1	UK	B.1	2022–05–15	Filipe,A. 2022
ON808414.1	UK	B.1	2022–05–15	Filipe,A. 2022
ON808415.1	UK	B.1	2022–05–15	Filipe,A. 2022
ON808416.1	UK	B.1	2022–05–15	Filipe,A. 2022

Accession number	Country	Lineage	Collection date	Reference
ON808417.1	UK	B.1	2022–05–15	Filipe,A. 2022
ON813251.2	Germany	B.1	2022–06	Brinkmann,A. 2022
ON813252.2	Germany	B.1	2022–06	Brinkmann,A. 2022
ON813253.2	Germany	B.1	2022–06	Brinkmann,A. 2022
ON813254.2	Germany	B.1	2022–06	Brinkmann,A. 2022
ON813255.2	Germany	B.1	2022–06	Brinkmann,A. 2022
ON813256.2	Germany	B.1	2022–06	Brinkmann,A. 2022
ON813257.2	Germany	B.1	2022–06	Brinkmann,A. 2022
ON813258.2	Germany	B.1	2022–06	Brinkmann,A. 2022
ON813259.2	Germany	B.1	2022–06	Brinkmann,A. 2022
ON813260.2	Germany	B.1	2022–06	Brinkmann,A. 2022
ON813261.2	Germany	B.1	2022–06	Brinkmann,A. 2022
ON813262.2	Germany	B.1	2022–06	Brinkmann,A. 2022
ON813263.2	Germany	B.1	2022–06	Brinkmann,A. 2022
ON813264.2	Germany	B.1	2022–06	Brinkmann,A. 2022
ON813265.2	Germany	B.1	2022–06	Brinkmann,A. 2022
ON813266.2	Germany	B.1	2022–06	Brinkmann,A. 2022
ON813267.2	Germany	B.1	2022–06	Brinkmann,A. 2022
ON838178.1	Slovenia	B.1	2022–06	Zakotnik,S. 2022
ON838939.1	Spain	B.1	2022–06–06	de la Hoz-Sanchez,B. 2022
ON838940.1	Spain	B.1	2022–05–20	de la Hoz-Sanchez,B. 2022
ON843163.1	Portugal	B.1	2022–05–13	Isidro,J. 2022
ON843164.1	Portugal	B.1	2022–05–18	Isidro,J. 2022
ON843165.1	Portugal	B.1	2022–05–24	Isidro,J. 2022
ON843166.1	Portugal	B.1	2022–05–24	Isidro,J. 2022
ON843167.1	Portugal	B.1	2022–05–26	Isidro,J. 2022
ON843168.1	Portugal	B.1	2022–05–26	Isidro,J. 2022
ON843169.1	Portugal	B.1	2022–05–26	Isidro,J. 2022
ON843170.1	Portugal	B.1	2022–05–26	Isidro,J. 2022
ON843171.1	Portugal	B.1	2022–05–26	Isidro,J. 2022
ON843172.1	Portugal	B.1	2022–05–27	Isidro,J. 2022
ON843173.1	Portugal	B.1	2022–05–27	Isidro,J. 2022
ON843174.1	Portugal	B.1	2022–05–30	Isidro,J. 2022
ON843175.1	Portugal	B.1	2022–05–30	Isidro,J. 2022
ON843176.1	Portugal	B.1	2022–06–02	Isidro,J. 2022
ON843177.1	Portugal	B.1	2022–06–02	Isidro,J. 2022
ON843178.1	Portugal	B.1	2022–06–02	Isidro,J. 2022
ON843179.1	Portugal	B.1	2022–06–02	Isidro,J. 2022
ON843180.1	Portugal	B.1	2022–06–03	Isidro,J. 2022
ON843181.1	Portugal	B.1	2022–06–06	Isidro,J. 2022
ON843182.1	Portugal	B.1	2022–05–30	Isidro,J. 2022
ON853649.1	Germany	B.1	2022–06	Brinkmann,A. 2022
ON853650.1	Germany	B.1	2022–06	Brinkmann,A. 2022
ON853651.1	Germany	B.1	2022–06	Brinkmann,A. 2022
ON853652.1	Germany	B.1	2022–06	Brinkmann,A. 2022
ON853653.1	Germany	B.1	2022–06	Brinkmann,A. 2022
ON853654.1	Germany	B.1	2022–06	Brinkmann,A. 2022
ON853655.1	Germany	B.1	2022–06	Brinkmann,A. 2022
ON853656.1	Germany	B.1	2022–06	Brinkmann,A. 2022
ON853657.1	Germany	B.1	2022–06	Brinkmann,A. 2022
ON853658.1	Germany	B.1	2022–06	Brinkmann,A. 2022
ON853659.1	Germany	B.1	2022–06	Brinkmann,A. 2022
ON853660.1	Germany	B.1	2022–06	Brinkmann,A. 2022
ON853661.1	Germany	B.1	2022–06	Brinkmann,A. 2022
ON853662.1	Germany	B.1	2022–06	Brinkmann,A. 2022
ON853663.1	Germany	B.1	2022–06	Brinkmann,A. 2022
ON853664.1	Germany	B.1	2022–06	Brinkmann,A. 2022
ON853665.1	Germany	B.1	2022–06	Brinkmann,A. 2022
ON853666.1	Germany	B.1	2022–06	Brinkmann,A. 2022
ON853667.1	Germany	B.1	2022–06	Brinkmann,A. 2022
ON853668.1	Germany	B.1	2022–06	Brinkmann,A. 2022
ON853669.1	Germany	B.1	2022–06	Brinkmann,A. 2022
ON853670.1	Germany	B.1	2022–06	Brinkmann,A. 2022
ON853671.1	Germany	B.1	2022–06	Brinkmann,A. 2022
ON853672.1	Germany	B.1	2022–06	Brinkmann,A. 2022
ON853673.1	Germany	B.1	2022–06	Brinkmann,A. 2022
ON853674.1	Germany	B.1	2022–06	Brinkmann,A. 2022
ON853675.1	Germany	B.1	2022–06	Brinkmann,A. 2022
ON853676.1	Germany	B.1	2022–06	Brinkmann,A. 2022

Accession number	Country	Lineage	Collection date	Reference
ON853677.1	Germany	B.1	2022–06	Brinkmann,A. 2022
ON853678.1	Germany	B.1	2022–06	Brinkmann,A. 2022
ON853679.1	Germany	B.1	2022–06	Brinkmann,A. 2022
ON853680.1	Germany	B.1	2022–06	Brinkmann,A. 2022
ON853681.1	Germany	B.1	2022–06	Brinkmann,A. 2022
ON853682.1	Germany	B.1	2022–06	Brinkmann,A. 2022
ON872184.1	Ireland	B.1	2022–05–30	Fletcher,N. 2022
ON880413.1	Brazil	B.1	2022–06–14	Coletti,T.M. 2022
ON880419.1	Belgium	B.1	2022–05–27	Wawina-Bokalanga,T. 2022
ON880420.1	Belgium	B.1	2022–05–27	Vanmechelen,B. 2022
ON880421.1	Belgium	B.1	2022–05–27	Vanmechelen,B. 2022
ON880422.1	Belgium	B.1	2022–05–27	Vanmechelen,B. 2022
ON880505.1	Canada	B.1	2022–05–20	Duggan,A. 2022
ON880506.1	Canada	B.1	2022–05–22	Duggan,A. 2022
ON880507.1	Canada	B.1	2022–05–27	Duggan,A. 2022
ON880508.1	Canada	B.1	2022–05–30	Duggan,A. 2022
ON880509.1	Canada	B.1	2022–05–29	Duggan,A. 2022
ON880510.1	Canada	B.1	2022–05–31	Duggan,A. 2022
ON880511.1	Canada	B.1	2022–05–30	Duggan,A. 2022
ON880512.1	Canada	B.1	2022–06–01	Duggan,A. 2022
ON880513.1	Canada	B.1	2022–06–04	Duggan,A. 2022
ON880514.1	Canada	B.1	2022–06–04	Duggan,A. 2022
ON880515.1	Canada	B.1	2022–06–02	Duggan,A. 2022
ON880516.1	Canada	B.1	2022–06–02	Duggan,A. 2022
ON880517.1	Canada	B.1	2022–05–27	Duggan,A. 2022
ON880518.1	Canada	B.1	2022–06–08	Duggan,A. 2022
ON880519.2	Canada	B.1	2022–05–13	Duggan,A. 2022
ON880520.1	Canada	B.1	2022–05–19	Duggan,A. 2022
ON880521.1	Canada	B.1	2022–05–19	Duggan,A. 2022
ON880522.1	Canada	B.1	2022–05–21	Duggan,A. 2022
ON880523.1	Canada	B.1	2022–05–20	Duggan,A. 2022
ON880524.1	Canada	B.1	2022–05–26	Duggan,A. 2022
ON880525.1	Canada	B.1	2022–05–25	Duggan,A. 2022
ON880526.1	Canada	B.1	2022–05–27	Duggan,A. 2022
ON880527.1	Canada	B.1	2022–05–27	Duggan,A. 2022
ON880528.1	Canada	B.1	2022–05–27	Duggan,A. 2022
ON880529.1	Canada	B.1	2022–05–28	Duggan,A. 2022
ON880530.1	Canada	B.1	2022–05–29	Duggan,A. 2022
ON880531.1	Canada	B.1	2022–05–29	Duggan,A. 2022
ON880532.1	Canada	B.1	2022–05–30	Duggan,A. 2022
ON880533.1	Canada	B.1	2022–05–30	Duggan,A. 2022
ON880534.1	Canada	B.1	2022–05–30	Duggan,A. 2022
ON880535.1	Canada	B.1	2022–05–31	Duggan,A. 2022
ON880536.1	Canada	B.1	2022–05–31	Duggan,A. 2022
ON880537.1	Canada	B.1	2022–05–31	Duggan,A. 2022
ON880538.1	Canada	B.1	2022–06–01	Duggan,A. 2022
ON880539.1	Canada	B.1	2022–06–01	Duggan,A. 2022
ON880540.1	Canada	B.1	2022–06–01	Duggan,A. 2022
ON880541.1	Canada	B.1	2022–06–01	Duggan,A. 2022
ON880542.1	Canada	B.1	2022–05–31	Duggan,A. 2022
ON880543.1	Canada	B.1	2022–06–02	Duggan,A. 2022
ON880544.1	Canada	B.1	2022–06–02	Duggan,A. 2022
ON880545.1	Canada	B.1	2022–06–02	Duggan,A. 2022
ON880546.1	Canada	B.1	2022–06–02	Duggan,A. 2022
ON880547.1	Canada	B.1	2022–06–03	Duggan,A. 2022
ON880548.1	Canada	B.1	2022–06–03	Duggan,A. 2022
ON880549.1	Canada	B.1	2022–06–03	Duggan,A. 2022
ON911481.2	Mexico	B.1	2022–06–28	Galan-Huerta,K.A. 2022
ON918656.1	Taiwan	B.1	2022–06	Lin,J.-H. 2022
ON927243.1	Spain	B.1	2022–06–21	Palomino-Cabrera,R. 2022
ON929057.1	Germany	B.1	2022–07	Brinkmann,A. 2022
ON929058.1	Germany	B.1	2022–07	Brinkmann,A. 2022
ON929059.1	Germany	B.1	2022–07	Brinkmann,A. 2022
ON929060.1	Germany	B.1	2022–07	Brinkmann,A. 2022
ON929061.1	Germany	B.1	2022–07	Brinkmann,A. 2022
ON929062.1	Germany	B.1	2022–07	Brinkmann,A. 2022
ON929063.1	Germany	B.1	2022–07	Brinkmann,A. 2022
ON929064.1	Germany	B.1	2022–07	Brinkmann,A. 2022
ON929065.1	Germany	B.1	2022–07	Brinkmann,A. 2022

Accession number	Country	Lineage	Collection date	Reference
ON929066.1	Germany	B.1	2022–07	Brinkmann,A. 2022
ON929067.1	Germany	B.1	2022–07	Brinkmann,A. 2022
ON929068.1	Germany	B.1	2022–07	Brinkmann,A. 2022
ON929069.1	Germany	B.1	2022–07	Brinkmann,A. 2022
ON929070.1	Germany	B.1	2022–07	Brinkmann,A. 2022
ON929071.1	Germany	B.1	2022–07	Brinkmann,A. 2022
ON929072.1	Germany	B.1	2022–07	Brinkmann,A. 2022
ON929073.1	Germany	B.1	2022–07	Brinkmann,A. 2022
ON929074.1	Germany	B.1	2022–07	Brinkmann,A. 2022
ON929075.1	Germany	B.1	2022–07	Brinkmann,A. 2022
ON929076.1	Germany	B.1	2022–07	Brinkmann,A. 2022
ON929077.1	Germany	B.1	2022–07	Brinkmann,A. 2022
ON929078.1	Germany	B.1	2022–07	Brinkmann,A. 2022
ON929079.1	Germany	B.1	2022–07	Brinkmann,A. 2022
ON929080.1	Germany	B.1	2022–07	Brinkmann,A. 2022
ON929081.1	Germany	B.1	2022–07	Brinkmann,A. 2022
ON929082.1	Germany	B.1	2022–07	Brinkmann,A. 2022
ON929083.1	Germany	B.1	2022–07	Brinkmann,A. 2022
ON929084.1	Germany	B.1	2022–07	Brinkmann,A. 2022
ON929085.1	Germany	B.1	2022–07	Brinkmann,A. 2022
ON929086.1	Germany	B.1	2022–07	Brinkmann,A. 2022
ON929087.1	Germany	B.1	2022–07	Brinkmann,A. 2022
ON929088.1	Germany	B.1	2022–07	Brinkmann,A. 2022
ON929089.1	Germany	B.1	2022–07	Brinkmann,A. 2022
ON929090.1	Germany	B.1	2022–07	Brinkmann,A. 2022
ON929091.1	Germany	B.1	2022–07	Brinkmann,A. 2022
ON950045.1	Belgium	B.1	2022–05–27	De Baetselier,I. 2022
ON954773.1	USA	B.1	2022–05	Gigante,C.M. 2022
ON959131.1	USA	B.1	2022–05	Gigante,C.M. 2022
ON959132.1	USA	B.1	2022–06	Gigante,C.M. 2022
ON959133.1	USA	B.1	2022–05	Gigante,C.M. 2022
ON959134.1	USA	B.1	2022–05	Gigante,C.M. 2022
ON959135.1	USA	B.1	2022–05	Gigante,C.M. 2022
ON959136.1	USA	B.1	2022–06	Gigante,C.M. 2022
ON959143.1	Finland	B.1	2022–06–16	Kant,R. 2022
ON959149.1	Germany	B.1	2022–07	Brinkmann,A. 2022
ON959150.1	Germany	B.1	2022–07	Brinkmann,A. 2022
ON959151.1	Germany	B.1	2022–07	Brinkmann,A. 2022
ON959152.1	Germany	B.1	2022–07	Brinkmann,A. 2022
ON959153.1	Germany	B.1	2022–07	Brinkmann,A. 2022
ON959154.1	Germany	B.1	2022–07	Brinkmann,A. 2022
ON959155.1	Germany	B.1	2022–07	Brinkmann,A. 2022
ON959156.1	Germany	B.1	2022–07	Brinkmann,A. 2022
ON959157.1	Germany	B.1	2022–07	Brinkmann,A. 2022
ON959158.1	Germany	B.1	2022–07	Brinkmann,A. 2022
ON959159.1	Germany	B.1	2022–07	Brinkmann,A. 2022
ON959160.1	Germany	B.1	2022–07	Brinkmann,A. 2022
ON959161.1	Germany	B.1	2022–07	Brinkmann,A. 2022
ON959162.1	Germany	B.1	2022–07	Brinkmann,A. 2022
ON959163.1	Germany	B.1	2022–07	Brinkmann,A. 2022
ON959164.1	Germany	B.1	2022–07	Brinkmann,A. 2022
ON959165.1	Germany	B.1	2022–07	Brinkmann,A. 2022
ON959166.1	Germany	B.1	2022–07	Brinkmann,A. 2022
ON959167.1	Germany	B.1	2022–07	Brinkmann,A. 2022
ON959168.1	Germany	B.1	2022–07	Brinkmann,A. 2022
ON959169.1	Germany	B.1	2022–07	Brinkmann,A. 2022
ON959170.1	Germany	B.1	2022–07	Brinkmann,A. 2022
ON959171.1	Germany	B.1	2022–07	Brinkmann,A. 2022
ON959172.1	Germany	B.1	2022–07	Brinkmann,A. 2022
ON959173.1	Germany	B.1	2022–07	Brinkmann,A. 2022
ON959174.1	Germany	B.1	2022–07	Brinkmann,A. 2022
ON959175.1	Germany	B.1	2022–07	Brinkmann,A. 2022
ON959176.1	Germany	B.1	2022–07	Brinkmann,A. 2022
ON959177.1	Germany	B.1	2022–07	Brinkmann,A. 2022
ON983159.1	Canada	B.1	2022–06–01	Duggan,A. 2022
ON983160.1	Canada	B.1	2022–06–03	Duggan,A. 2022
ON983161.1	Canada	B.1	2022–06–03	Duggan,A. 2022
ON983162.1	Canada	B.1	2022–06–03	Duggan,A. 2022
ON983163.1	Canada	B.1	2022–06–07	Duggan,A. 2022

Accession number	Country	Lineage	Collection date	Reference
ON983164.1	Canada	B.1	2022–06–09	Duggan,A. 2022
ON983165.1	Canada	B.1	2022–06–14	Duggan,A. 2022
ON983166.1	Canada	B.1	2022–06–15	Duggan,A. 2022
ON983167.1	Canada	B.1	2022–06–16	Duggan,A. 2022
ON983168.1	Czech Republic	B.1	2022–04–28	Chmel,M. 2022
OX009124.1	Sweden	B.1	2022–05–22	Pettke,A. 2022

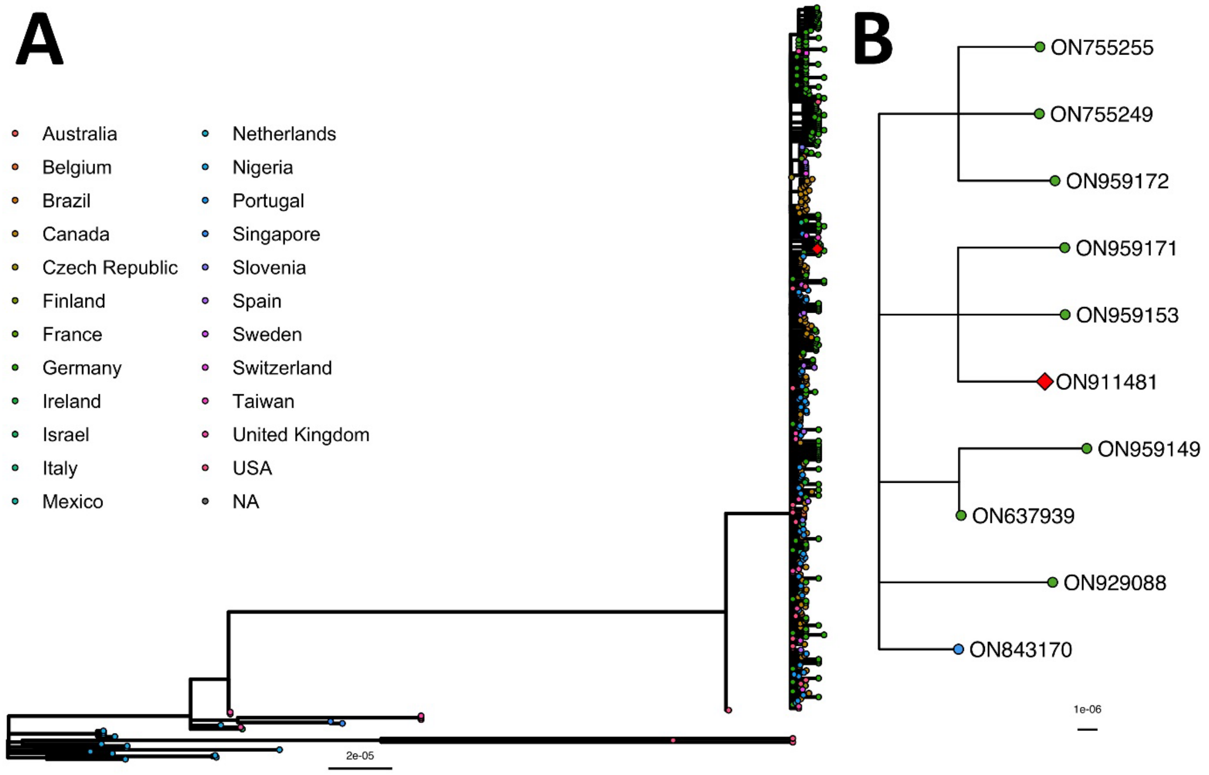

Appendix Figure 1. A) Phylogenetic relationship of hMPXV1 obtained from Nextstrain. B) Clade indicating the Mexican isolate and related sequences. The red diamond corresponds to the sequence from this study.

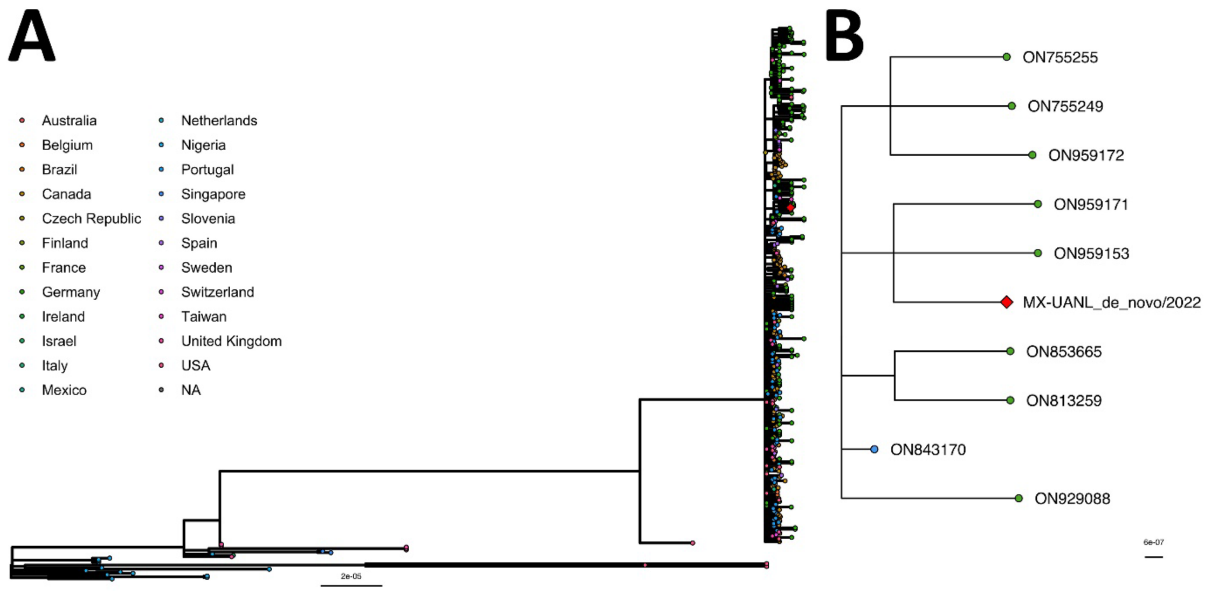

Appendix Figure 2. A) Phylogenetic relationship of hMPXV1 obtained from Nextstrain with de-novo assembly. B) Clade indicating the Mexican isolate and related sequences. The red diamond corresponds to the sequence from this study.
